# Fatherhood contributes to increased hippocampal spine density and anxiety regulation in California mice

**DOI:** 10.1002/brb3.416

**Published:** 2015-12-01

**Authors:** Erica R. Glasper, Molly M. Hyer, Jhansi Katakam, Robyn Harper, Cyrus Ameri, Thomas Wolz

**Affiliations:** ^1^ Department of PsychologyUniversity of MarylandCollege ParkMaryland20742; ^2^ Program in Neuroscience and Cognitive ScienceUniversity of MarylandCollege ParkMaryland20742

**Keywords:** Anxiety, dendritic spines, fatherhood

## Abstract

**Introduction:**

Parenting alters the hippocampus, an area of the brain that undergoes significant experience‐induced plasticity and contributes to emotional regulation. While the relationship between maternal care and hippocampal neuroplasticity has been characterized, the extent to which fatherhood alters the structure and function of the hippocampus is far less understood.

**Methods:**

Here, we investigated to what extent fatherhood altered anxiety regulation and dendritic morphology of the hippocampus using the highly paternal California mouse (*Peromyscus californicus*).

**Results:**

Fathers spent significantly more time on the open arms of the elevated plus maze, compared to non‐fathers. Total distance traveled in the EPM was not changed by paternal experience, which suggests that the increased time spent on the open arms of the maze indicates decreased anxiety‐like behavior. Fatherhood also increased dendritic spine density of granule cells in the dentate gyrus and basal dendrites of pyramidal cells in area CA1 of the hippocampus.

**Conclusions:**

These findings parallel those observed in maternal rodents, suggesting that the hippocampus of fathers and mothers respond similarly to offspring.

## Introduction

Fatherhood‐induced changes to the brain are not well understood, in large part due to the small number of paternally behaving mammals. The California mouse (*Peromyscus californicus)* is an excellent rodent model in which to study parenting, as males and females of this biparental species care for offspring similarly (Dudley 1974). This species provides a rare opportunity to study the effects of paternal care on brain plasticity. While few studies exist, available evidence suggests that males and females of biparental species experience similar changes to the structure of the brain—especially the hippocampus. The hippocampus has received much attention because of continued structural modifications (i.e., adult neurogenesis; spinogenesis) throughout adulthood and because of the role it plays in emotional regulation, cognition, and stress reactivity—all of which are altered during the postpartum period of maternal rodents. The paternal brain may undergo similar changes, however, it has been far less studied.

As previously mentioned, hippocampal structural morphology is significantly altered during the postpartum period in mothers (reviewed in Leuner et al. 2010). Rodent mothers experience reduced adult neurogenesis (Leuner et al. 2007; Pawluski and Galea 2007; Glasper et al. 2011) and increased dendritic spine density in the hippocampus (Kinsley et al. 2006; Pawluski and Galea 2006; Leuner and Gould 2010). Given that both adult neurogenesis (Clelland et al. 2009; Jessberger et al. 2009) and changes in dendritic spines (Leuner and Shors 2013) may underlie hippocampal function, it is not surprising that emotional regulation is also altered during the postpartum period in mothers. Specifically, maternal rodents exhibit reduced anxiety‐like behavior on the elevated plus maze (EPM) during the postpartum period (Lonstein 2005)—an effect that is dependent on offspring interaction. Interestingly, some fatherhood‐induced alterations to the hippocampus have also been observed. Fatherhood decreases new neuron survival in monogamous voles (Lieberwirth et al. 2013) as well as in California mouse fathers (Glasper et al. 2011), however, this change in adult neurogenesis is not accompanied by altered hippocampal function 1 month following the birth of pups (Glasper et al. 2011). To date, the extent to which fatherhood alters dendritic morphology of the hippocampus, and whether this change is associated with anxiety regulation, has yet to be investigated.

## Materials and Methods

Gonadally intact male, gonadally intact female, and tubally ligated female California mice (60–90 days old) were obtained from the Peromyscus Genetic Stock Center (University of South Carolina, Columbia, SC) or bred in our colony. Mice were provided ad libitum access to food and water, and were maintained on a reversed 16:8 light/dark cycle (lights off at 11:00 h). Two groups were used: non‐fathers (*n* = 8) and fathers (*n* = 12). Non‐fathers were gonadally intact virgin males that were pair housed with tubally ligated females, while fathers were gonadally intact virgin males that were pair housed with gonadally intact virgin females. Paired mice cohabitated on average 51.1 days before an average of 1.6 pups were born. Four fathers had a second litter present at the time of euthanization. Two litters were born on the same day of euthanization, 1 litter was born 24 h prior to euthanization, and 1 litter was present for nearly 1 week before euthanization.

A 5‐min EPM task was used to assess anxiety‐like behavior, as previously described in the California mouse (Chauke et al. 2012). Testing occurred on PND19, during a time of peak pup retrieval in California mouse fathers (Bester‐Meredith et al. 1999). Behavior was observed 2 h after lights out, under red light illumination, and monitored by EthoVision^®^ XT behavioral tracking software (Noldus, Leesburg, VA). Percent time spent in the open arms, total distance traveled, and number of arm entries was calculated. In the event that a mouse fell off of the EPM (*n* = 3), they were quickly placed into the center of the maze and allowed to explore the maze until a total of 5 min had passed. Heat maps were generated to represent the average location on the maze for each group. Warmer colors indicated more, while cooler colors represented less, time spent in a location of the maze. Three non‐fathers and 1 father were excluded from analysis due to >40% immobility during testing (Chauke et al. 2012). These excluded mice remained completely immobile for extended periods of time on the open or center arms of the maze and therefore were eliminated from the study. At the conclusion of EPM testing, mice were returned to their home cages and remained undisturbed until weaning on PND35.

On PND35, mice were deeply anesthetized, cervically dislocated, and brains were quickly harvested and processed for Golgi impregnation using a Rapid Golgi Staining Kit (FD Neurotechnologies, Columbia, MD), as previously described (Haim et al. 2015).

Dendritic remodeling analyses were performed, as previously described (Glasper et al. 2010), using a Zeiss AxioImager microscope with a stage controller and neuroimaging software (Neurolucida, Williston, VT). Five neurons per brain and five dendrites per neuron were used to assess spine density, dendritic length, and dendritic branching from randomly selected Golgi‐impregnated cells throughout the entire rostral‐caudal extent of the dentate gyrus (DG) and area CA1 of the hippocampus.

Data were analyzed using Prism 6.0 Software for Mac OSX (GraphPad Software Inc., San Diego, CA). Unpaired Student's t‐tests were performed to assess the effects of fatherhood on anxiety‐like behavior and hippocampal structural morphology. Pearson correlations were performed, where appropriate. Mean differences were statistically different when *P* ≤ 0.05.

## Results

**Figure 1:**
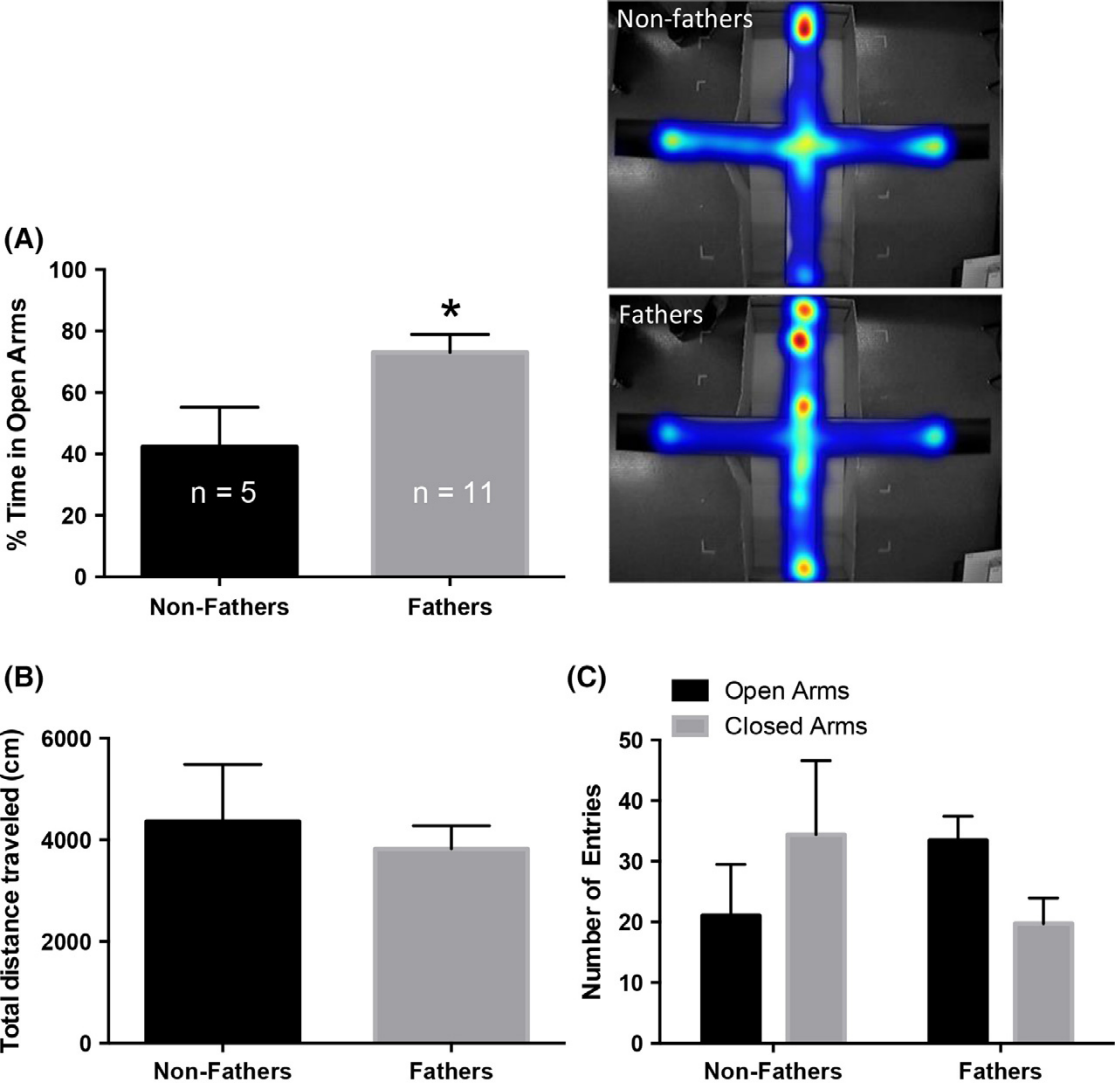
Fatherhood decreases anxiety‐like behavior in California mice. (A) Fatherhood increases the percent time spent in the open arms of the elevated plus maze (EPM), compared to non‐fathers. Heat maps indicate the average location of mice. Warmer colors represent more, while cooler colors represent less, time spent in a location on the EPM. Greater heat is observed on the open arm of the elevated plus maze in fathers. (B) No difference in the total distance traveled within the EPM was observed. (C) The number of entries into the open or closed arms did not differ between groups. Bars represent mean+SEM. *P ≤ 0.05.

Fatherhood significantly decreased anxiety‐like behavior on the EPM. Increased percent time spent exploring the open arms of the EPM was observed among fathers, compared to non‐fathers (*t* (14) = 2.53, *P* ≤ 0.05; Fig. 1A). No differences in the total distance traveled within the EPM were observed (*P* > 0.05; Fig. 1B). Open and closed arm entries did not differ between groups (*P* > 0.05; Fig. 1C). Due to increased freezing behavior, three non‐fathers were excluded from the analyses. This likely increased the variance in open arm entries that may have prevented non‐fathers and fathers from being statistically different from each other.

**Figure 2:**
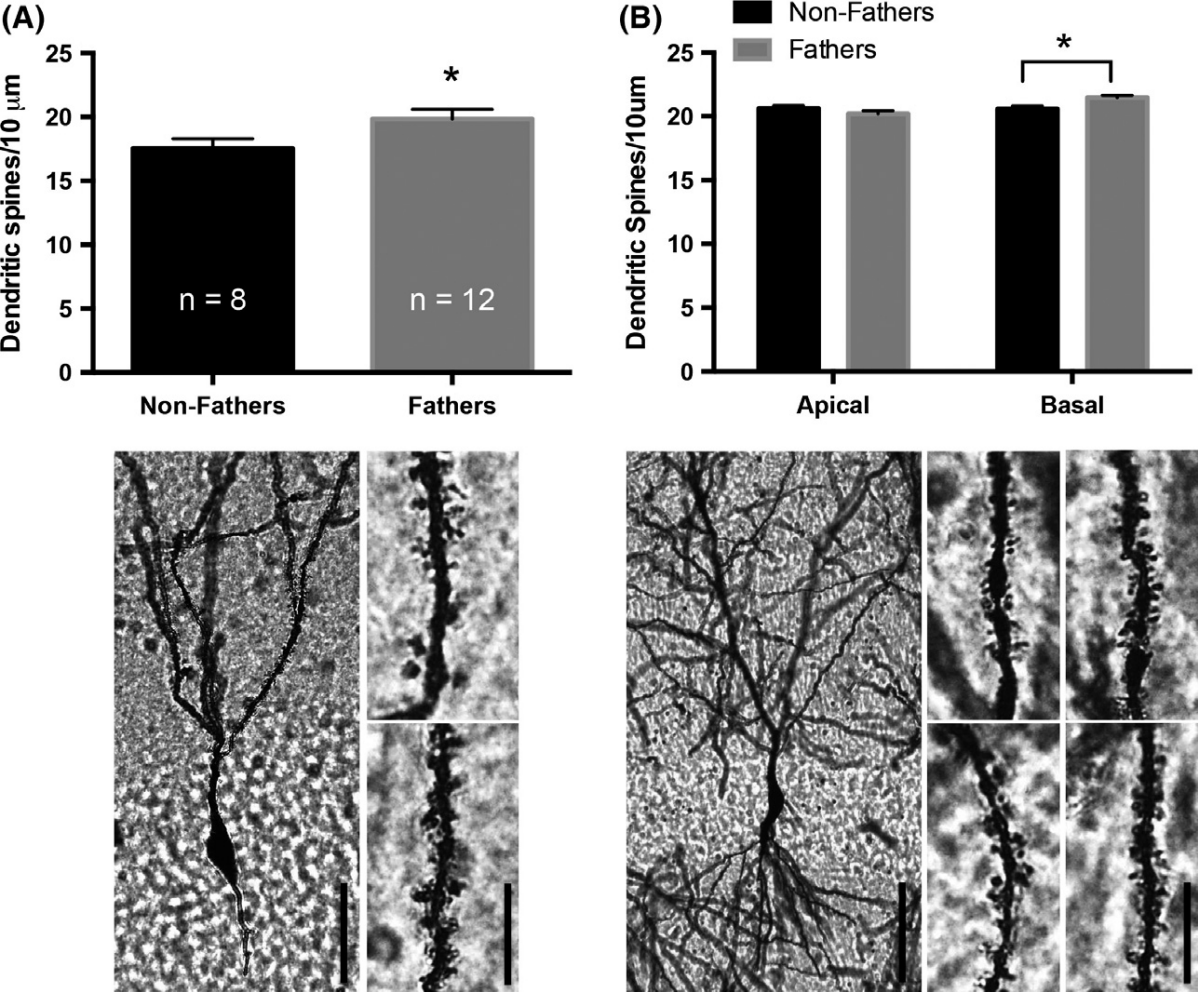
Fatherhood increases hippocampal dendritic spine density. (A, top) Fatherhood increases dendritic spine density of granule cell neurons in the dentate gyrus (DG). (A, bottom) The photomicrograph on the left depicts a representative DG granule cell from a male California mouse. Dendritic segments on the right are representative of non‐fathers (top) and fathers (bottom). (B, top) Dendritic spine density on basal, but not apical, dendrites of CA1 pyramidal cells is increased with fatherhood. (B, bottom) The photomicrograph on the left depicts a representative CA1 pyramidal cell from a male California mouse. Dendritic segments represent non‐fathers (left), fathers (right), apical (top), and basal (bottom). Bars represent mean+SEM. Scale bars: cells = 40 *μ*m, segments = 10 *μ*m. *P ≤ 0.05.

Fatherhood increased dendritic spine density. Increased dendritic spine density on secondary and tertiary dendrites was observed on DG granule cells (*t* (18) = 2.099, *P* ≤ 0.05; Fig. 2A), while dendritic length and the number of branch points were not altered by paternal experience (*P* > 0.05). Basal dendritic spine density of pyramidal cells within area CA1 of the hippocampus was increased by fatherhood (*t* (18) = 2.831, *P* ≤ 0.05; Fig. 2B), however, no change in dendritic spine density was observed on pyramidal cell apical dendrites within area CA1 (*P* > 0.05). CA1 pyramidal cell basal dendritic tree lengths (non‐father: 697.5 ± 50.80; father: 639.0 ± 68.36) and number of branch points (non‐father: 7.20 ± 0.638; father: 5.567 ± 0.669) were not different (*P* > 0.05). However, fathers had significantly shorter CA1 pyramidal cell apical dendritic tree lengths (*t* (18) = 2.615, *P* ≤ 0.05; non‐father: 845.9 ± 48.8; father: 638.2 ± 55.86) and fewer branch points (*t* (18) = 2.752, *P* ≤ 0.05; non‐father: 8.175 ± 0.284; father: 5.817 ± 0.668).

Performance on the elevated plus maze did not correlate with spine density in the DG or area CA1 of the hippocampus (*P* > 0.05).

## Discussion

This study examined the effects of fatherhood on hippocampal plasticity in California mice, a biparental species that forms strong bonds with both their mate and offspring (Gubernick and Nordby 1993). Our results suggest that fatherhood alters the structure and function of the hippocampus—a brain region that undergoes significant experience‐induced plasticity. The hippocampus plays an important role in the regulation of anxiety (Kheirbek et al. 2013). Parenting‐induced enhancements in anxiety regulation have been shown in maternal rodents (Lonstein 2005). We demonstrate that fatherhood decreases anxiety‐like behavior during a period of time when pup retrieval is elevated in fathers of this species (Bester‐Meredith et al. 1999). This offspring‐induced decrease in anxiety‐like behavior, among fathers, is similar to that observed in maternal rodents—an effect that is independent of suckling (Lonstein 2005). This suggests that pup contact, and not a mechanism related to lactation, may indeed drive these observed effects in fathers. However, not all biparental male rodents demonstrate enhanced anxiety regulation with paternal experience. Male prairie voles (*Microtus ochrogaster*) exhibit increased anxiety‐like behavior on the EPM a few days following the birth of offspring (Lieberwirth et al. 2013). Timing of behavioral testing may be the key to understanding these discordant observations. Starting at birth, California mouse fathers interact with their offspring by demonstrating huddling and licking behaviors that are followed by a surge in pup retrievals that occurs between PND15 and PND21 (Bester‐Meredith et al. 1999). We may have observed a different anxiety profile had we measured performance on the EPM earlier during the postpartum period. Interestingly, new California mouse fathers and paired virgins exhibit decreased anxiety‐like behavior on PND 3–4 when compared to isolated virgins and expectant fathers (Chauke et al. 2012). An analysis of the development of emotional regulation in fathers of this species should be carefully investigated.

Here, we demonstrate for the first time that fatherhood increases dendritic spine density of DG granule cells and basal CA1 pyramidal cells—an observation previously seen in maternal rodents (Kinsley et al. 2006; Pawluski and Galea 2006; Leuner and Gould 2010; Salmaso et al. 2011). Collectively, these data suggest that the effects of offspring on parenting‐induced hippocampal plasticity are similar between sexes. It is important to note that enhanced dendritic plasticity in fathers has been observed in the prefrontal cortex of the biparental marmoset (*Callithrix jacchus*; Kozorovitskiy et al. 2006) and in the medial precentral cortex of California mice (Kozorovitskiy 2007). The hippocampus was not assessed in these previous studies. Our study also observed dendritic atrophy of apical dendrites of CA1 pyramidal cells in fathers, compared to non‐fathers. Chronic stress has been reported to reduce dendritic complexity of CA1 neurons in rats (Donohue et al. 2006; Pawluski and Galea 2006). We do not know whether California mouse males find fatherhood stressful (i.e., activation of the HPA axis). The degree to which fatherhood alters the stress response and its differential effects on region‐specific hippocampal structural plasticity is unknown.

Available evidence from fathers of biparental species suggests that paternal care is influenced through direct father–offspring contact (Dixson and George 1982; Bredy et al. 2004). Increased offspring contact improves memory (Aguggia et al. 2013), decreases anxiety (Maniam and Morris 2010), and decreases depression (Boccia et al. 2007) in maternal rodents, however, the extent to which altered offspring contact (i.e., separation from offspring) changes hippocampal function in fathers is unknown. It is likely that offspring contact may be neuroprotective in species where strong pair bonds exist between parents and offspring during the postpartum period. Direct comparisons between species that demonstrate different social structures could shed an interesting light on the role of bonding in parenting‐induced neuroplasticity.

The mechanisms responsible for fatherhood‐induced changes in neuroplasticity are unknown, however, many of the observed changes in neuroplasticity in maternal rodents are due to alterations in circulating hormones (Lucas et al. 1998; Darnaudéry et al. 2007; Brusco et al. 2008). Although paternal rodents do not undergo pregnancy, parturition, or lactation, interaction with offspring has been shown to alter hormone concentrations (for a comprehensive review, see Saltzman and Ziegler 2014). One such hormone that is altered with offspring interaction is prolactin (PRL). Higher PRL concentrations are observed in California mouse fathers 2d postpartum, compared to virgin males or expectant fathers (Gubernick and Nelson 1989) and PRL concentrations in marmoset fathers are higher than in males without offspring (Dixson and George 1982). To date, the direct effects of PRL on hippocampal dendritic spine density, in fathers of any species, is unknown. However, given PRL's role in mediating offspring contact, and its likely role in other forms of offspring‐induced structural plasticity (i.e., adult neurogenesis; Mak and Weiss 2010; Lévy et al. 2011), it is possible that PRL plays a role in our observed findings. Another potential contributor to our fatherhood‐induced changes in hippocampal neuroplasticity is oxytocin (OT). By PND3, California mouse fathers have higher OT concentrations than fathers separated from their offspring on PND0 (Gubernick et al. 1995), suggesting that bond formation may contribute to alterations in OT concentrations in this species. While direct manipulation of OT and its effects on hippocampal neuroplasticity in males is unknown, administration of OT to nulliparous women increases functional connectivity within the hippocampus during presentation of infant‐related cues (Riem et al. 2012), suggesting that OT may contribute to altered neuroplasticity of the hippocampus in parents. Finally, given that vasopressin (AVP) has been associated with offspring‐induced alterations to structural plasticity in marmoset fathers (Kozorovitskiy et al. 2006), it is likely a candidate here. AVP correlates with paternal care in California mouse fathers (Bester‐Meredith and Marler 2003) and AVP gene expression is elevated in prairie vole fathers (Wang et al. 2001).

In conclusion, these results demonstrate that the postpartum period in fathers is a time of significant plasticity within the hippocampus. DG structural morphology and anxiety regulation are enhanced during a time of peak pup interaction in fathers. These data suggest that interaction with offspring may influence mood and structural changes within the brain of fathers as it does in maternal rodents. However, whether the changes in hippocampal structure underlie the observed behavioral change is still unknown and should be explored. Additionally, future studies should investigate whether maternal California mice also exhibit similar modifications to DG neuroplasticity. Taken together, these novel data increase our knowledge of paternal experience‐induced plasticity and raise interesting questions about the mechanisms driving these observed effects.

## Conflict of Interest

None declared.
